# Evaluation of Tooth Erosion Potential of Zero and Carbonated Beverages in Korea: A Laboratory‐Based Study Focused on pH

**DOI:** 10.1111/idh.70069

**Published:** 2026-04-16

**Authors:** Eun‐Ha Jung, Hyun‐Kyung Yim, Ji‐Hyun Min

**Affiliations:** ^1^ Department of Dental Hygiene, College of Science and Technology Kyungpook National University Sangju Korea; ^2^ Department of Dental Hygiene, College of Medical Convergence Catholic Kwandong University Korea; ^3^ Department of Dental Hygiene, College of Health and Medical Sciences Cheongju University Cheongju Korea

**Keywords:** beverages, low calories, sugar substitute, zero calories

## Abstract

**Objectives:**

With increasing health consciousness, the consumption of zero‐calorie beverages (zero beverages) is also increasing. Zero beverages are devoid of sugar or alternative sugar content. Tooth erosion possibility continues to be reported in cases of carbonated beverage consumption. However, studies evaluating the likelihood of tooth erosion from carbonated beverages containing alternative sugars are lacking. Therefore, this study assessed the tooth erosion ability of carbonated beverages distributed in South Korea.

**Methods:**

After purchasing 135 types of commercially available beverages in Korea, according to the Ministry of Food and Drug Safety's standards, those with less than 5 kcal were classified as carbonated zero beverages (CBzero), and those with more than 4 kcal per 100 mL were classified as typical carbonated beverages (CBnon‐zero). The experimental beverages were measured in 50 mL quantities at 25°C. An experienced investigator immediately measured the pH of the beverages using a pH meter. The pH between groups was compared using the Mann–Whitney *U* test in SPSS statistics version 27.0 analysis program.

**Results:**

Among the beverages included, 62 (average pH of 2.97 ± 0.36) and 73 (average pH was 3.19 ± 0.63) were in the CBnon‐zero and CBzero groups, respectively. Differences between the groups were not significant.

**Conclusions:**

This study demonstrated that the mean pH of zero beverages was not significantly different from that of regular carbonated beverages available in the Korean market. Given that the majority of these beverages exhibited a pH below 4.0, both CBzero and CBnon‐zero drinks may pose a comparable risk for dental erosion. Therefore, consumers should be aware that zero beverages, despite their reduced sugar content, are not exempt from potential erosive effects on teeth.

## 1 Introduction

The global carbonated beverage market will predictably exhibit a Compound Annual Growth Rate of 6.5% by 2028 [1]. According to a study examining Korea's beverage consumption market, since 2018, coffee accounted for 24.2%, followed by carbonated beverages at 22%, with higher consumption than bottled water (15.2%) and fruit and vegetable beverages (12.2%) [2].

According to the Ministry of Food and Drug Safety standards for each food type, carbonated beverages are carbonated beverages or water containing carbon dioxide gas [3]. Recently, along with increased interest in health and weight control, the consumption of ‘zero’ foods, in which sugars such as sugar in processed foods are replaced with alternative sweeteners, is increasing worldwide [4]. Zero beverages generally refer to beverages with zero‐ or very‐low‐calorie content. According to the U.S. Code of Federal Regulations, beverages can be labelled as zero if they contain less than 5 kcal per serving [5]. Korea's Ministry of Food and Drug Safety allows the sale of beverages as zero beverages if they contain less than 4 kcal per 100 mL or less than 5 kcal per serving [6]. Starting with the launch of Coca‐Cola Zero and Nadrang Cider in 2006, Korea has shown a rapid increase in sales of zero beverages since 2021 [2, 7]. Alternative sweeteners are used instead of sugars to manufacture zero‐beverages [8]. Alternative sweeteners used in zero food include aspartame, sucralose, and acesulfame K. These have a sweet taste and do not provide any or reduce calories, which helps with weight loss and are also used by diabetic patients who need to control their blood sugar levels [9]. In addition, in cases such as xylitol, a reduction in dental caries and tooth erosion can be expected owing to the use of alternative sweeteners as a mechanism to prevent dental caries [10].

Meanwhile, carbonated beverages contain sugar for sweetness and acid for a refreshing feeling, which reportedly affects dental health and high tooth erosion because of their low pH [11]. Therefore, the possibility of tooth erosion due to zero beverages consumption with adjusted sugar content using an alternative sweetener cannot be exempted. Furthermore, even carbonated water without any sugar content causes tooth erosion [12].

Although numerous studies have investigated the erosive potential of carbonated beverages, limited data are available regarding the pH characteristics of zero‐sugar beverages available in Korea. Therefore, in this study, we evaluated the tooth erosion potential of zero beverages by comparing the pH of carbonated and zero beverages using alternative sweeteners currently sold at supermarkets and convenience stores in Korea and to provide information on this effect, examined the potential impact of zero beverage consumption on teeth among zero‐beverage consumers.

## 2 Materials and Methods

### 2.1 Data Collection and Classification

This study was conducted in accordance with the Preferred Reporting Items for Laboratory Experiments (PRILE) 2021 guidelines [13]. This study did not require ethical approval, as it involved no human or animal subjects and only included analysis of commercially available beverages. We purchased manufacturer‐presented carbonated beverages for two days (July 20, 2023, to July 21, 2023). In order to secure product diversity and representativeness, beverages were collected from three major supermarkets (E‐Mart, Homeplus, and Lotte Mart) and three major convenience stores (CU, 7‐Eleven, and GS25) located in Suwon‐si, Gyeonggi‐do. All beverages available for purchase in the domestic market were included in the study, regardless of their country of manufacture. No random allocation or concealment was applicable, as no experimental intervention was performed. All available beverage products that met the inclusion criteria (carbonated and labelled calorie content) were included to ensure representativeness. According to the ‘Food Labeling Regulation’ of the Korean Food and Drugs Administration, if calories in the product are less than 4 per 100 mL, the product can be labelled as ‘0 kcal’ [6]. Based on these criteria, we divided the beverages into a group of carbonated beverages containing 4 or below 4 kcal (CBzero) and another group beverages containing over 4 kcal (CBnon‐zero).

### 2.2 pH Measurement

We used an Orion^TM^ 3‐Star Benchtop pH Meter (Thermo Fisher Scientific, Waltham, MA, USA) to measure the pH of each beverage. Before measurement, the pH meter was calibrated using standard buffer solutions of pH 4.0 and 7.0. A researcher measured the pH three times for each 50 mL product immediately after opening the product cap at 25°C. Since this study involved objective pH measurements using a calibrated digital pH meter, blinding of the operator or outcome assessors was not applicable. However, to minimize measurement bias, all measurements were conducted by a single trained examiner under standardized conditions. To intuitively present the erosive potential of each beverage on dental enamel, the beverages were categorized based on their pH levels as follows: extremely erosive (pH < 3.0), erosive (pH < 4.0), and minimally erosive (pH ≥ 4.0).

### 2.3 Statistical Analysis

We recorded the pH data as range and mean (standard deviation [SD]). The normality between CBzero and CBnon‐zero was confirmed using the Shapiro–Wilk test, and the mean difference in pH values between the two groups was determined using the Mann–Whitney *U* test. All analyses were performed using the SPSS Statistics ver. 27.0 (IBM Corp., Armonk, NY, USA). The level of statistical significance was *p* < 0.05.

## 3 Results

In this study, we classified 135 beverages. Sixty‐two beverages were included in the CBnon‐zero group, with a mean pH of 2.97 ± 0.36 (Table 1). Among these, 32 beverages (51.6%) were classified as extremely erosive (pH < 3.0), and 29 beverages (46.8%) were classified as erosive (pH < 4.0). Seventy‐three beverages were included in the CBzero, with a mean pH of 3.19 ± 0.63 (Table 2). Within this group, 35 beverages (47.9%) were classified as extremely erosive, and 30 beverages (41.1%) were classified as erosive. The difference in the mean pH between the CBnon‐zero and CBzero groups was not statistically significant (*p* = 0.114; Table 3).

**TABLE 1:** Group of carbonated beverages containing over 4 kcal per 100 mL (*n* = 62).

Product name	kcal/100 mL^†^	pH (Standard deviation)
Extremely erosive		
Bundaberg ginger beer	45.3	2.89 (0.01)
Bundaberg lemon lime & bitters	45.3	2.62 (0.01)
Canada dry ginger ale	32	2.71 (0.01)
Chilsung cider grape	42.4	2.75 (0.01)
Chilsung cider plus	6	2.90 (0.01)
Coca cola original taste	44	2.34 (0.05)
Coolpis talk sparkling pineapple	44.11	2.92 (0.04)
Demisoda lemon	34	2.64 (0.07)
Demisoda apple	34	2.95 (0.01)
Dr. pepper	41.69	2.43 (0.02)
Fanta orange	27.2	2.54 (0.03)
Fanta pineapple	20.8	2.82 (0.01)
Franklin elderflower lemonade	18.18	2.65 (0.00)
Franklin rose lemonade	31.27	2.74 (0.01)
Galvanina grapefruit	40.56	2.77 (0.02)
Hot6 the king rush	4.2	2.90 (0.01)
Korean zombie energy drink	38	2.47 (0.04)
Korean zombie energy drink low calorie	4.2	2.59 (0.04)
Kwangdong on the game	27.5	2.98 (0.01)
Loux lemon juice drink	48.5	2.52 (0.16)
Loux orange juice drink	48.5	2.87 (0.12)
Miero fibre sparkling zero	11.4	2.55 (0.14)
Mountain dew	47.9	2.81 (0.03)
Oran C—pineapple low calorie	19.6	2.74 (0.01)
Oronamin c drink	58.3	2.83 (0.01)
Paris cola	36.36	2.70 (0.01)
Pepsi	44.00	2.36 (0.04)
Pitapop	8.00	2.97 (0.00)
Puyo soda – soda flavour	42.86	2.43 (0.10)
Sunkist sparkling lemonade	52.00	2.39 (0.05)
Sunkist sparkling grapefruit	50.70	2.80 (0.01)
Vita 500 sparkling fizzy lime	50.00	2.82 (0.01)
Erosive		
Bundaberg pink grapefruit	46.6	3.08 (0.01)
Chilsung cider	42.1	3.02 (0.02)
Coolpis talk sparkling peach	44.11	3.35 (0.02)
Demisoda white grape	34	3.04 (0.08)
Demisoda peach	34	3.18 (0.01)
Fanta milk soda	52.4	3.47 (0.01)
Franklin orange & grapefruit	33.09	3.49 (0.02)
Galvanina blood orange	43.94	3.07 (0.02)
Hot6	47.9	3.05 (0.05)
Hot6 the king force	5.4	3.16 (0.01)
Hot6 the king punch	26	3.15 (0.01)
Hot6 the king power	47.8	3.15 (0.00)
Kwangdong on the green	18.8	3.23 (0.01)
Mccol	47.4	3.45 (0.04)
Milkis	52	3.40 (0.01)
Monster energy	50.7	3.55 (0.01)
Monster energy mango loco	49.3	3.32 (0.01)
Monster energy pipeline punch	37.2	3.03 (0.00)
Monster energy zero suger	4.5	3.62 (0.01)
Ocean spray sparkling peach cranberry	57.4	3.37 (0.01)
Orangina	41.7	3.13 (0.03)
Protein sparkling grape	29.17	3.24 (0.01)
Protein sparkling pink grapefruit	27.08	3.28 (0.07)
Red bull energy drink	46.00	3.47 (0.01)
Red bull red edition	44.00	3.13 (0.01)
Schweppes lemon tonic	38.86	3.01 (0.02)
Sprite	45.60	3.07 (0.01)
Welch's grape	51.83	3.02 (0.02)
Yogurt sparkling	13.00	3.02 (0.21)
Minimally erosive		
Apple trevi plus	6.0	4.20 (0.01)

^†^ kcal/100 mL values are presented as labeled on each product; decimal places were not standardized as values varied across products.

**TABLE 2:** Group of carbonated beverages containing 4 or below 4 kcal per 100 mL (*n* = 73).

Product name	kcal/100 mL^†^	pH (Standard deviation)
Extremely erosive		
All at me v sparkling	2.57	2.59 (0.01)
Baskin robbins apple mint zero	1.14	2.11 (0.12)
Baskin robbins rainbow sherbet sparkling zero	1.14	2.52 (0.01)
Celsius sparkling orange flavour	3.07	2.80 (0.13)
Celsius sparkling Fuji apple pear	3.07	2.20 (0.16)
Chilsung cider zero blue lime	0.00	2.99 (0.01)
Coca cola zero	0.00	2.59 (0.02)
Coca cola zero zero	0.00	2.76 (0.03)
Cloop zero soda green apple	0.00	2.70 (0.03)
Cloop zero soda pineapple	0.00	2.20 (0.18)
Cloop zero soda grape	0.00	2.75 (0.03)
Cloop zero soda peach	0.00	2.55 (0.13)
Colite grape pop	0.00	2.95 (0.01)
Dr. pepper zero	0.00	2.99 (0.01)
Dunkin zero sparkling pear	0.00	2.74 (0.03)
Dunkin zero sparkling plum	0.00	2.69 (0.01)
Famous soda co pink lemonade	2.42	2.84 (0.04)
Konga gaseosa	0.25	2.61 (0.10)
Narangd cider zero	0.00	2.96 (0.20)
Narangd cider zero pineapple	0.00	2.88 (0.02)
One am sparkling orange	0.00	2.71 (0.01)
Rupee's zero sparkling energy tart cherry	2.90	2.94 (0.01)
Singha lemon soda	0.00	2.72 (0.20)
Sprite zero	0.00	2.85 (0.04)
Sugarlolo sparkling apple	0.00	2.83 (0.01)
Sugarlolo sparkling shine muscat	0.00	2.91 (0.02)
Tabcaa original	2.82	2.62 (0.01)
Tams zero apple × kiwi flavour	1.41	2.97 (0.02)
Tams zero orange flavour	2.25	2.41 (0.11)
Tams zero white grape × pomegranate flavour	2.25	2.94 (0.08)
Vitamin c lemon sparkling	2.00	2.46 (0.06)
Vtalk zero lemonade	0.00	2.54 (0.01)
Welch's zero grape	0.00	2.90 (0.02)
Welch's zero shine muscat	0.00	2.40 (0.08)
Welch's zero orange	0.00	2.39 (0.11)
Erosive		
Chilsung cider zero	0.00	3.01 (0.00)
Hot6 zero	3.20	3.17 (0.02)
Hot6 the king zero	2.30	3.17 (0.03)
Mccol zero	2.00	3.61 (0.03)
Milkis zero	3.20	3.45 (0.01)
Monster energy ultra	3.9	3.59 (0.01)
Monster energy ultra citron	3.1	3.18 (0.00)
Monster energy ultra paradise	3.9	3.54 (0.01)
Monster energy ultra sunrise	2.8	3.49 (0.00)
Mont best with sparkling water lemon	0	3.83 (0.01)
Mont best with sparkling water lime	0	3.80 (0.08)
Narangd cider zero green apple	0.00	3.11 (0.05)
One am sparkling lime	0.00	3.21 (0.01)
Pepsi zero sugar mango flavour	0.00	3.05 (0.01)
Pepsi zero sugar lime flavour	0.00	3.23 (0.02)
Redbull sugar free	2.00	3.53 (0.01)
Seagram's plain	0.00	3.70 (0.05)
Seagram's lime	0.00	3.80 (0.11)
Seagram's lemon	0.00	3.85 (0.02)
Sugarlolo clear cola	0.00	3.76 (0.01)
Sugarlolo sparkling lemon cider	0.00	3.14 (0.01)
Sugarlolo sparkling peach	0.00	3.07 (0.01)
Tams zero lemon flavour	2.25	3.16 (0.02)
Tams zero lemon peach × passion fruit flavour	2.25	3.01 (0.03)
Victoria white grape	0.00	3.75 (0.01)
Victoria kiwi	0.00	3.78 (0.07)
Victoria pear	0.00	3.74 (0.07)
Victoria pineapple	0.00	3.76 (0.04)
Vida original citrus zero	0.00	3.19 (0.02)
Vida salty lychee zero	0.00	3.03 (0.01)
Minimally erosive		
ChoJung sparkling water	0.00	4.20 (0.17)
ChoJung sparkling water lemon	0.00	4.44 (0.16)
Trevi grapefruit	0.00	4.50 (0.02)
Trevi lemon	0.00	4.32 (0.06)
Trevi lime	0.00	4.53 (0.02)
Trevi peach	0.00	4.19 (0.01)
Trevi plain	0.00	4.51 (0.04)
Perrier lime	0.00	5.10 (0.08)

^†^ kcal/100 mL values are presented as labeled on each product; decimal places were not standardized as values varied across products.

**TABLE 3:** Differences in pH depending on the kilocalories per 100 mL.

Groups	*N*	Mean pH (standard deviation)	*p*
CBnon‐zero	62	2.97 (0.36)	0.114
CBzero	73	3.19 (0.63)	
Total mean (standard deviation)		3.09 (0.53)	

## 4 Discussion

Tooth erosion is a multifactorial disease due to biological, behavioural, and chemical factors [14, 15]. Among the chemical factors, not only the pH of acids in foods and beverages but also other properties—such as buffer capacity and the concentrations of calcium and phosphate—play critical roles in determining the severity of erosion. Beverages like yogurt, despite having low pH values (pH 3.7–4.3), do not contribute to dental erosion because they cause saliva to become supersaturated with calcium and phosphate ions [15, 16]. In contrast, flavoured waters containing citric acid may also exhibit low pH levels, but due to their low buffer capacity, they are rapidly neutralized by saliva and therefore pose minimal erosive risk [15, 16]. Moreover, the erosive potential of a beverage can vary depending on the type of acid it contains and its degree of dissociation [17]. Organic acids such as citric, malic, and lactic acids, which differ in their pKa values and buffering capacities, exert different effects on enamel dissolution [17]. These findings emphasize the need for a multifactorial chemical assessment when evaluating the erosive potential of beverages [14, 15, 16, 17].

Among several chemical factors, the pH remains one of the most widely studied and easily measurable indicators of erosive potential [18]. The pH scale represents the hydrogen ion concentration, calculated as log10[H^+^]. In a previous study attempting to calculate and predict the depth of enamel loss caused by commercial drinks through enamel loss prediction equations using pH and titratable acidity, pH was found to be a more critical indicator than titratable acidity in determining the extent of enamel erosion [19]. This suggests that pH may still serve as a key indicator in dental erosion research.

According to a previous study by Larsen et al. [18], beverages with a pH of 4.00 or lower evidently developed tooth erosion and erosion was minimized in beverages with a pH of 4.20 or higher. A previous study by Lussi et al. [14] confirmed that tooth surface microhardness was significantly reduced in beverages and foodstuffs with a pH below 4.00. Of the 135 commercially available carbonated beverages in Korea surveyed in this study, only nine had a pH of 4.00 or higher, and the remaining 126 had a pH range of 2.11 to 3.85. Their overall average pH was 3.09 ± 0.53. A previous study examining pH for various commercial beverages in the United States found that 84 had a pH range of 2.32–3.88, and 10 had a pH range of 4.00–5.24 [20]. Their overall average pH was 3.12 ± 0.53 [20]. Therefore, most carbonated beverages in the Korean and the United States markets had a pH below 4.00, facilitating tooth erosion [18].

In this study, the groups were divided based on 4 kcal per 100 mL of beverage to investigate the difference in the average pH of the low‐ and high‐calorie beverages. Sucrose, fructose, glucose, and other simple sugars are used as energy sources by bacteria in dental plaques and release organic acids as metabolites, resulting in dental caries [21]. Sugar substitutes are food additives that mimic the effect of sugar on taste, and saccharin, aspartame, sucralose, sorbitol, mannitol, xylitol, maltitol, erythritol, and lactitol are commonly added as sugar substitutes in commercial beverages, are non‐ or low‐calorie sweetening agents and are not fermented by microorganisms in the oral cavity; therefore, they do not cause dental caries [22]. Therefore, sugar substitution is beneficial regarding dental caries occurrence.

However, it differs regarding tooth erosion caused by chemical acids without the involvement of microorganisms in the oral cavity. Carbonic acid is an aqueous carbon dioxide solution obtained by injecting CO_2_ into H_2_O, with the molecular formula H_2_CO_3_. As it decomposes, H^+^ ions are produced, which play a role in lowering the pH of carbonated drinks. According to a systematic review of adolescents' dietary habits, 52% of 42 studies showed that carbonated drinks were significantly and positively associated with tooth erosion [23]. A systematic review of the enamel damage due to carbonated drinks concluded that the pH and exposure period of carbonated drinks adversely affected the enamel [24]. Therefore, the possibility of carbonic acid‐caused tooth erosion warrants caution. Moreover, as a result of this study, the average pH of the CBnon‐zero and CBzero groups were 2.97 ± 0.36 and 3.19 ± 0.63, respectively, with no statistically significant difference (*p* = 0.114). Therefore, a low‐calorie intake does not guarantee low tooth erosion. Given the increasing popularity of zero‐calorie carbonated beverages in South Korea, especially among health‐conscious consumers, it is important to note that these beverages are not necessarily safer for dental health in terms of acidity [3]. Despite having no or low sugar content, many zero‐calorie beverages still exhibit low pH levels, posing a potential risk for tooth erosion.

A limitation of this study is that only pH was investigated among the various properties that can predict the possibility of tooth erosion in carbonated drinks. In addition, its application is limited in real‐life situations or clinical practice because the experiment was not conducted by applying it to natural teeth. Therefore, further studies should evaluate not only additional chemical properties such as buffer capacity and mineral content but also the actual erosive potential in intraoral conditions. Nonetheless, investigating the pH levels of a wide range of commercially available carbonated beverages in South Korea represents a foundational contribution to understanding their erosive potential.

## 5 Conclusion

This study demonstrated that the mean pH of zero beverages was not significantly different from that of regular carbonated beverages available in the Korean market. Given that the majority of these beverages exhibited a pH below 4.0, both CBzero and CBnon‐zero drinks may pose a comparable risk for dental erosion. Therefore, consumers should be aware that zero beverages, despite their reduced sugar content, are not exempt from potential erosive effects on teeth.

## 6 Clinical Relevance

### 6.1 Scientific Rationale of the Study

Overall, 135 carbonated drinks in the Korean market were collected and classified into zero and non‐zero beverages, and their pH was investigated in this study.

### 6.2 Principal Findings

Most of the 135 carbonated drinks had a pH of 4.00. The 73 drinks were classified as zero drinks. The average pH between the zero‐ and non‐zero‐beverage groups was not statistically significant.

### 6.3 Practical Implications

Consumers should be aware that zero‐carbonated drinks, which have recently become popular, may cause tooth erosion and should be careful when consuming them.

## Author Contributions

J.‐H.M. conceived the ideas; E.‐H.J. and H.‐K.Y. planned methods for generating the results; J.‐H.M., E.‐H.J., and H.‐K.Y. performed the statistical analysis. J.‐H.M. led the writing; E.‐H.J., H.‐K.Y. and J.‐H.M. approved the final manuscript for publication. We agree to take responsibility for all aspects of this manuscript.

## Funding

The authors have nothing to report.

## Conflicts of Interest

The authors declare no conflicts of interest.

## Data Availability

The data that support the findings of this study are available from the corresponding author upon reasonable request.
